# A Case of Complicated Traumatic Generalized Surgical Emphysema, Pneumomediastinum, Pneumopericardium, Pneumothorax, and Pneumoperitoneum Due to Accidental Dislodgement of Tracheostomy Tube

**DOI:** 10.7759/cureus.20762

**Published:** 2021-12-27

**Authors:** Hany A Zaki, Adel Zahran, Abdallah M Elsafti Elsaeidy, Ahmed E Shaban, Eman E Shaban

**Affiliations:** 1 Emergency Medicine, Hamad Medical Corporation, Doha, QAT; 2 Internal Medicine, Mansoura General Hospital, Mansoura, EGY; 3 Mansoura University, Faculty of Medicine, Mansoura, EGY; 4 Cardiology, Al Jufairi Diagnostic and Therapeutic Hospital, Doha, QAT

**Keywords:** accidental decannulation, pneumothorax (ptx), invasive mechanical ventilation, tracheal tube, pneumomediastinum, tracheostomy

## Abstract

A tracheostomy tube (TT) is usually taken out in a well-planned and coordinated manner after the underlying condition that necessitated the procedure is resolved. The inadvertent removal or dislodgement of the TT from the stroma is known as accidental extubation or decannulation. This event may prove fatal in a stable patient. Like other respiratory procedures, tracheostomy with the long-term placement of tracheal tube comes with several risks, including scarring of the trachea, pneumothorax, tracheal rupture, and tracheoesophageal fistula. Other complications may include pneumomediastinum (PM) or the escape of air into the surrounding tissue. This may be attributed to several reasons, including mispositioning of the tracheal tube, barotrauma, or tracheal rupture. In some cases, PM presents with free air into cavities such as the thorax, peritoneum, or subcutaneous tissue. Although not fatal, it may require complex treatments such as ventilator management, high-flow oxygen, or, in some cases, surgical intervention. In this article, we describe a rare case of PM and generalized surgical emphysema due to mispositioning of the tracheal tube.

## Introduction

A tracheostomy tube is usually placed for several reasons. It may be done to bypass an obstruction of the upper airway or due to inability to wean from mechanical ventilation, failure to contain excessive secretions, and impaired neurologic status [1-3]. The tracheostomy tube, when placed, facilitates the patient's transfer from the intensive care unit (ICU) to a weaning facility, which may be either a long-term care hospital or a step-down unit [4]. It is important to note that a tracheostomy is used only as a short-term requirement for patients and should be removed once its use is completed.

Prolonged presence of tracheostomy tubes may bring up complications such as tracheal and subglottic stenosis, formation of granulation tissue, depression and rupture of the tracheal wall, and tracehoinnominate fistulas [5]. Pneumomediastinum (PM), also known as mediastinal emphysema, refers to the presence of free air in the cavity of the mediastinum. The etiology of PM may be a direct result of trauma, or it may have an iatrogenic cause due to endoscopic procedures or other therapeutic procedures [6]. The condition may also be spontaneous in a physiologic process setting or other chronic pulmonary conditions [7]. PM presents independently or, based on the etiology, may present alongside pneumoperitoneum and other manifestations of free air [8]. In this article, we present a rare case of PM and generalized surgical emphysema due to mispositioning of the tracheal tube in a 75-year-old male patient.

## Case presentation

A 75-year-old-male patient with a history of type II diabetes mellitus, old stroke, hypertension, dementia, intertrochanteric fracture of left femur, and surgical history of the gamma nail fixation under spinal anesthesia presented to our emergency “resuscitation room” referred from long-term facility by emergency medical services after experiencing a sudden swelling of the face, lip, and tongue, followed by generalized body swelling. The cause was traced to suspected angioedema triggered by a new fungal medication (anidulafungin). The referring facility had prescribed epinephrine, hydrocortisone, and diphenhydramine for him without any improvement.

Upon initial examination, the patient was found to have hypoactive delirium. He was placed on a tracheostomy with mechanical ventilation. Venous blood gas test showed acidosis with high pCO_2_.

Investigations showed that the tracheostomy tube had accidentally moved from its position due to the patient’s movements. The patient also suffered a recent episode of acute cholecystitis and sepsis and was treated conservatively with antibiotics.

Vital signs as observed were as follows: temp axillary - 36.1 C; heart rate peripheral - 85 bpm, respiratory rate - 18 breaths/min, systolic blood pressure - 112 mmHg, diastolic blood pressure - 72 mmHg, SpO_2_ - 100%.

Physical examination of the patient revealed an apyrexial appearance, absence of jaundice, anemia, clubbing, cyanosis, or lymphadenopathy. There was no neck rigidity or rash. The patient was also bed-ridden.

Examination of the head and neck showed severe puffiness and edema on both eyes, large, protruded tongue with both lips swelling.

Examination of the cardiovascular system showed a regular pulse, normal character, jugular venous pressure not raised, and heart sounds with first and second, and no murmurs. Examination of the respiratory system showed palpable diffuse chest crepitus without skin rashes. There was no exertion of breathing. Chest normally expanded, while percussion notes were resonant. Chest sounds were equal and bilateral. There were no added sounds, and SpO_2_ was 100% on room air.

Examination of the gastrointestinal (GI)/genitourinary (GU) system and abdominal region revealed a distended and relatively rigid abdomen. There was no organomegaly and no masses. Bowel sounds were normal, while hernial orifices were intact.

Examination of the extremities showed mild pitting edema on both ankles.

**Figure 1:**
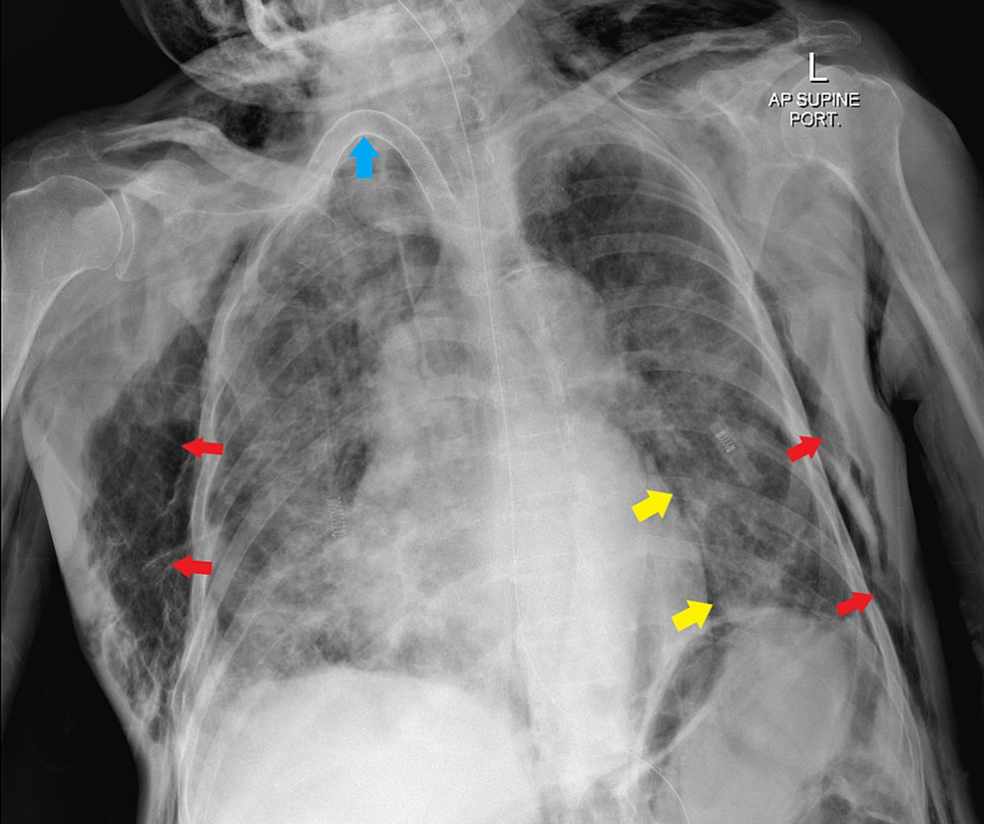
Newly developed surgical emphysema involving the chest walls, more on the right side as well as the root of the neck bilaterally (red arrows). There is also suspicion of pneumomediastinum, especially on the left side (yellow arrows). Tracheostomy tube and nasogastric tubes are noted (blue arrow). Redemonstrations of the previously described bilateral pulmonary patchy heterogeneous opacities. Both costophrenic angles are minimally blunted.

X-ray of the chest showed severe generalized surgical emphysema and PM. There was an immediate consult to the ENT, medical intensive care unit, anesthesia, and thoracic surgeon. Figure 1 shows chest X-ray.

Examination under fiberoptic scope showed the mucosa overhanging the end of the tracheostomy tube. This resulted in a narrow opening and, hence, high ventilatory pressure. The distal trachea was normal up to the carina. The ENT surgeon railroaded a 12.3 cm tracheostomy tube using a bougie but was again stuck at the same level. Fiberscope was passed beyond the obstruction, and the tube passed successfully beyond the obstruction. Ventilatory pressures then dropped to acceptable levels with very good tidal volumes. The anesthetist was on the board as intubation might be needed in case of failed tracheostomy tube insertion.

Laboratory results: creatinine 276 - previously creatinine was normal, high inflammatory markers, C-reactive protein trending up, procalcitonin >100, leukocytosis, arterial blood gas test showed respiratory acidosis, pH 7.069, lactate 0.8, pCO_2_ 70, and bicarbonate 20.

**Figure 2:**
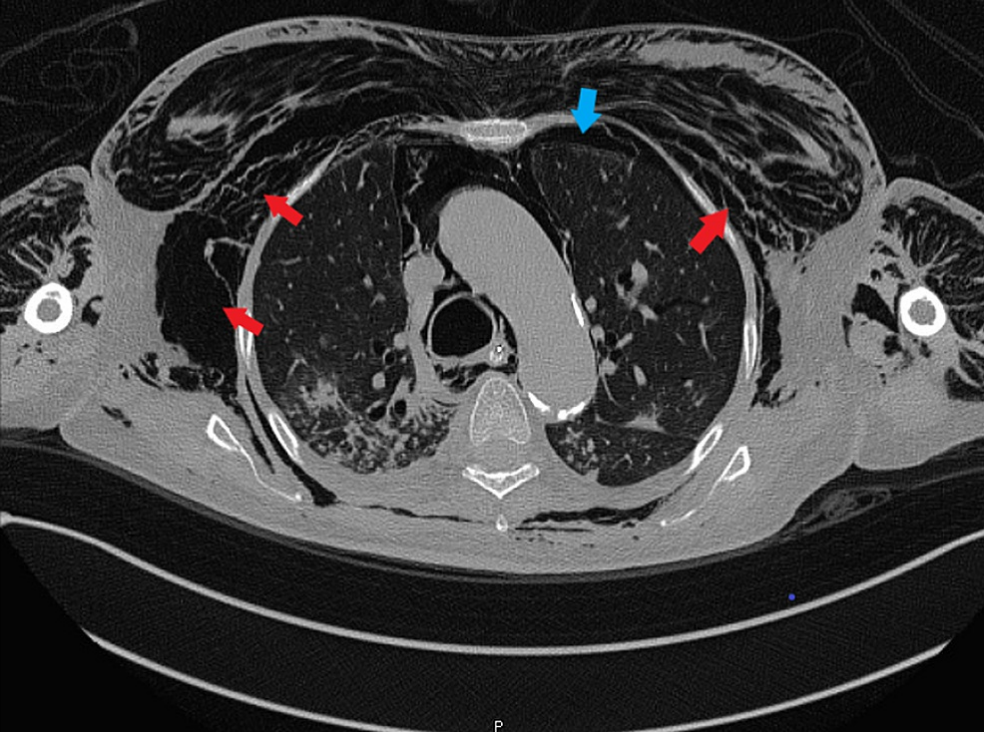
Computed tomography axial view of the chest demonstrates bilateral massive surgical emphysema mainly on the right side (red arrows), with evidence of left-sided pneumothorax (blue arrow).

Computed tomography (CT) imaging examination: The evaluation was limited due to motion artifact and extensive emphysema. A tracheostomy tube was identified, with its distal tip impinging upon the posterior wall of the trachea. An extensive PM, including pneumopericardium, small left-sided pneumothorax, and extensive chest wall emphysema, tracking to the partially visualized soft tissues of bilateral upper limbs was found (Figure 2).

**Figure 3:**
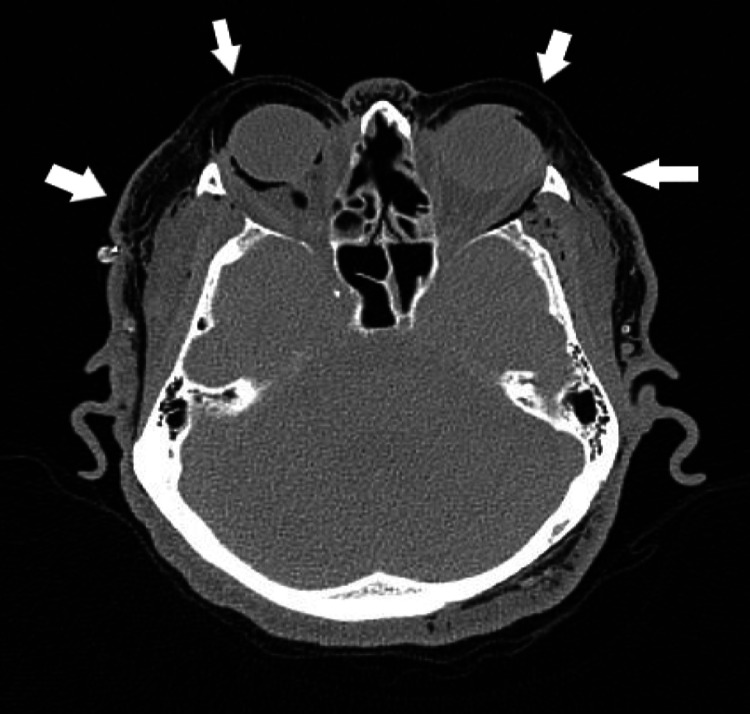
Computed tomography axial view of the head demonstrates extensive emphysema in almost all of the compartments of the visualized neck (white arrows). We also saw bilateral intra-orbital emphysema.

Extensive emphysema was identified in almost all of the compartments of the visualized neck. We also saw bilateral intra-orbital emphysema (Figure 3).

**Figure 4:**
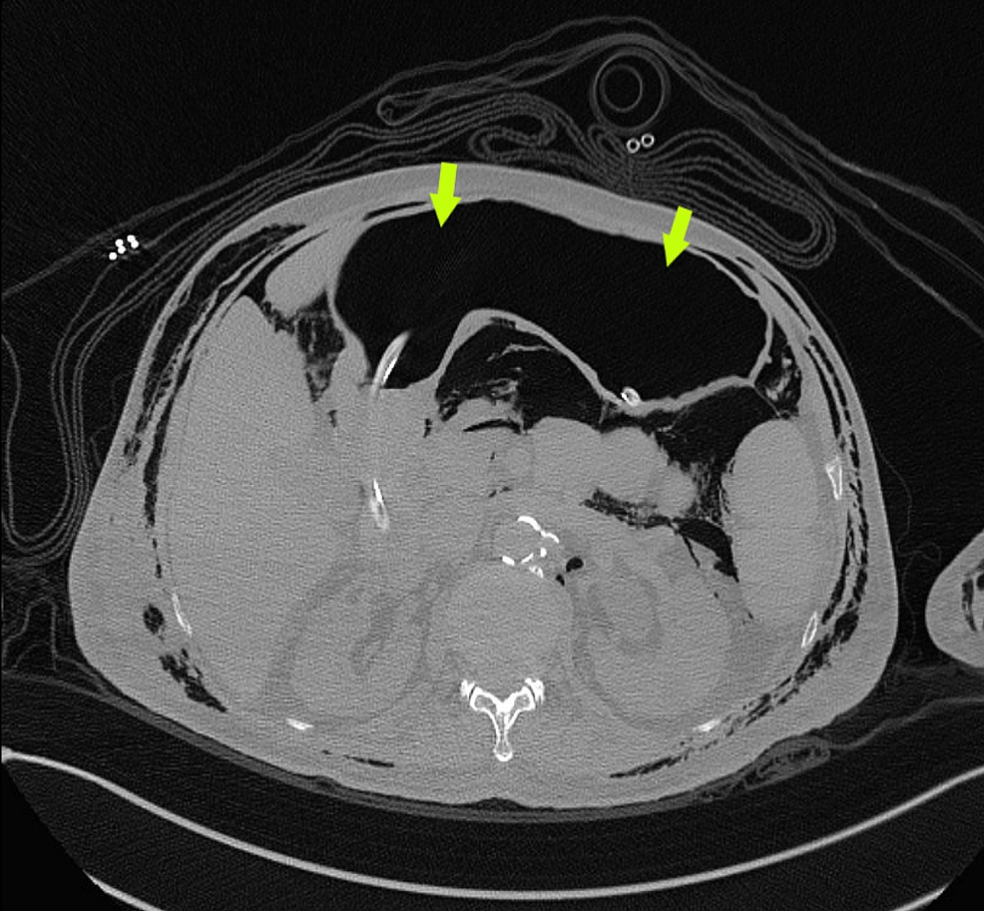
Computed tomography axial view of the abdomen (light green arrows) demonstrates massive pneumoperitoneum.

There is the presence of multisegmented nodular opacities, atelectasis and consolidation, and surrounding ground-glass opacities in both lungs with predominance in the posterior segments. Right-sided pleural fluid was minimal. We also observed bilateral scattered cystic bronchiectasis. Also, the CT abdomen showed a massive pneumoperitoneum (Figure 4).

Management/follow-up plan: The medical team reviewed the patient with the following plan: continuation with Tazocin while considering resumption of anidulafungin as the patient mainly had emphysema, and not angioedema. Continue antibiotic therapy as per infectious disease (ID) team plan, and follow sepsis workup. Regarding kidney injury/oliguria: conservative treatment, monitor inflammatory markers, avoid nephrotoxic medications, and monitor renal function tests. The case was discussed with the family with risks and benefits of dialysis being explained, and dialysis was refused by the family.

Regarding the current general status of the patient, dialysis may not be the best option now. We will consider conservative management and reevaluate the patient response, avoiding hypotension and nephrotoxic medications. 

The ID specialist reviewed the patient. They reviewed positive blood cultures. Blood cultures taken from the central line grew yeast-like cells. Keeping into consideration that the patient might have an allergic reaction to the anidulafungin, we might consider changing to alternate antifungals. Follow final cultures. Remove/change lines if in situ >10 days. Will start liposomal amphotericin empirically. Stop teicoplanin. Continue Tazocin for now. Adjust/de-escalate according to available cultures and patients’ clinical conditions and progress.

**Figure 5:**
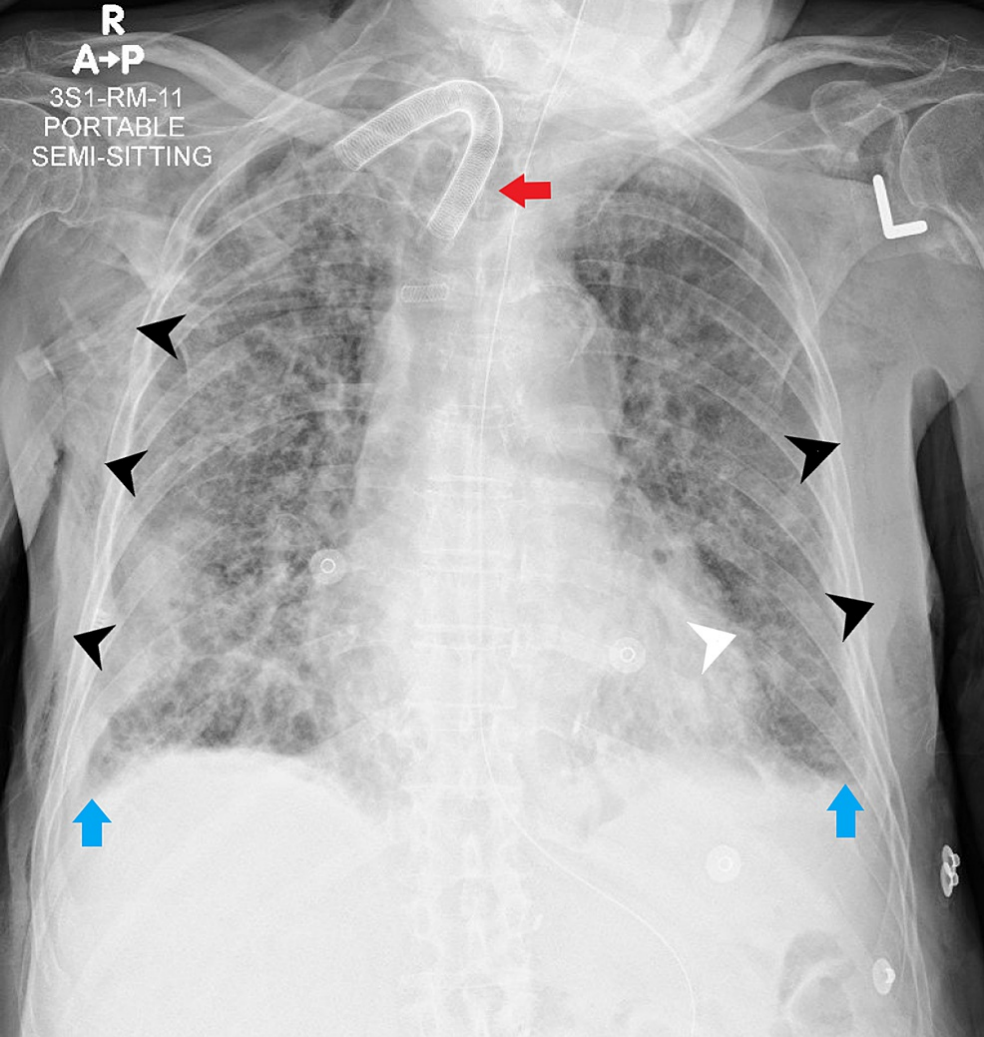
Chest X-ray one week after hospital admission showing significant improvement of the surgical emphysema involving the chest walls (black arrowheads), tracheostomy tube, and a nasogastric tube (red arrow). Redemonstrations of the previously described bilateral pulmonary patchy heterogeneous opacities. Minimally blunted costophrenic angles (blue arrows), and significant improvement of the pneumopericardium (white arrowhead).

Internal medicine reviewed the patient again: The patient was not communicating, but emphysema improved (Figure 5).

Urine output measured 1.4 L, and family requested for percutaneous endoscopic gastrostomy. The patient was reviewed again with the thoracic surgeon and was advised to continue the conservative treatment with observation and there is no need for a chest tube for now unless there is a clinical deterioration, an increase in O_2_ requirement, or an increase in the size of the pneumothorax on imaging. The patient was already septic with low urine output and high inflammatory markers two days before transferring due to acute exacerbation on top of chronic cholecystitis, complicated with oliguric acute kidney injury (now anuric). Lasix infusion was started for 24 hours but there was no improvement in urine output.

Dialysis was initiated through a femoral catheter, and the output was clear yellow urine from the catheter. The patient was seen later in the afternoon again awake, moving arms, and more alert than in the morning. 

## Discussion

This article describes the case of a 75-year-old man diagnosed with pneumoperitoneum, PM, and generalized subcutaneous emphysema attributed to the mispositioning of a tracheal tube. Based on the report by family members and physical examination, it appears that our patient may have suffered some form of mechanical insult resulting in entry of free air into the subcutaneous tissue, peritoneum, and mediastinum. From the imaging, we could not determine the precise location of the insult or origin of air penetration. Generally, complications associated with tracheostomies depend heavily on the time from which the procedure took place. Early complications are linked to the immediate surgery, including injury to the tracheal wall, tube dislodgement, subcutaneous emphysema, and obstruction of the tracheal tube [5]. Late complications are common and may occur in at least 60% of patients. These complications may include tube dislodgement, mispositioning, subcutaneous emphysema, or tracheal stenosis [9]. Also, injury to the wall of the trachea from wrongful positioning of the tube or high cuff pressure may cause ulceration and creation of fistulas with closer structures like tracheoinnominate artery fistula and tracheoesophageal fistula [9]. Mispositioning of the tracheostomy tube is a complication that occurs late and may manifest in up to 10% of patients [10]. The most common malposition is tracheostomy tube occlusion by the posterior wall of the trachea, which has been observed in at least 92% of cases [10].

This contrasts with tracheostomy dislodgement, which is a fatal event with up to 50% mortality rate [11]. Termination of the tracheal tube should be 2-3 cm above the carina, just like endotracheal tubes. With regard to the anatomy, the trachea is passing through the mediastinum at this depth. If the tracheal wall is disrupted at this location, then there is a high risk of air leaking into the space of the mediastinum, ultimately resulting in PM, as seen in our patient.

There is a dichotomy in the etiology of PM, one being a result of blunt force trauma (which is the cause in our patient) or having an iatrogenic cause due to endoscopy or other therapeutic procedures. Another cause is the presence of free air in the cavity of the mediastinum with no clear etiology [6]. There are reports of subcutaneous emphysema occurring in at least 92% of PM cases, as seen in our patient [12]. There was also a substantial occurrence of pneumoperitoneum illustrated by the spaces lying between the mediastinum, pneumoperitoneum, and subcutaneous tissue being anatomically continuous, and emergence of air from a lesion in these areas may flow to another region along fascial planes [13].

For such patients to be clinically examined, the clinician must have adequate knowledge of the precise location and presence of the free air. Our patient had abundant subcutaneous emphysema, which was reflected in the physical examination. In spontaneous versions of PM, only 18% of patients showed evidence of Hamman’s crunch [6]. Upon examination of our patient, we found evidence of palpable diffuse chest crepitus. It is important to note that the crepitus commonly occurs in subcutaneous emphysema and is self-limiting [14]. Examination of the GI/GU and abdominal system revealed that our patient’s abdomen was distended in the pneumoperitoneum setting. PM, in many cases, is usually self-limiting if not associated with alimentary tract perforation. There is the risk of abdominal compartment syndrome, which may be assessed via the determination of bladder pressure. PM usually involves common complaints such as neck pain, dyspnea, chest pain, rhinolalia, and cough [6].

The location of free air in the body determines its treatment. There was a multifocal presence of free air in the peritoneum, mediastinum, and subcutaneous tissue in our patient. Pneumothorax, mediastinal emphysema, and subcutaneous emphysema are usually reabsorbed into the capillaries via diffusion along a partial pressure gradient due to the sum of partial pressures exerted by oxygen, nitrogen, carbon dioxide, and water. When breathing pure oxygen, nitrogen washes out of the blood, leading to an increase in the gradient for gas absorption and resulting in a four- to six-fold increase in gas absorption rate [15]. Ventilatory support also contributes to efficient management. While there is no set positive end-expiratory pressure (PEEP) setting or tidal volume, these parameters must be readjusted to very low levels that can be tolerated while also achieving optimum gas exchange as high PEEP can cause barotrauma complications as pneumothorax, extra-thoracic dissection, and pneumothorax [16]. Subcutaneous emphysema has also been addressed by other methods. Some studies have shown that subcutaneous air may be eliminated by a 2-3 cm blowhole incision in the infraclavicular or supraclavicular area. This may be somewhat challenging for patients placed on ventilators as mechanical ventilation usually results in the formation of air in large amounts. Severe subcutaneous emphysema may be treated with negative pressure wound therapy alongside blowhole incisions [17].

The complications of PM depend on the etiology. Complications are extremely rare in the setting of spontaneous PM due to the presence of a self-limiting course. Traumatic or iatrogenic PM may be linked with other pathologies like pneumopericardium or pneumothorax with a slight risk of converting to 'malignant' or tension PM, resulting in great vessel compression [18].

## Conclusions

Tracheal and tracheostomy tubes are widely used in patients requiring extended ventilatory support. Though they are well-practiced and routine techniques, they still have a high risk of complications. Our patient required mechanical ventilatory support, no doubt, but we understand that tracheal mispositioning may have played a major role in causing the pneumoperitoneum, PM, and subcutaneous emphysema.
